# Synchrotron micro-X-ray fluorescence imaging of arsenic in frozen-hydrated sections of a root of *Pteris vittata*

**DOI:** 10.1093/mtomcs/mfab009

**Published:** 2021-03-09

**Authors:** Teruhiko Kashiwabara, Nobuyuki Kitajima, Ryoko Onuma, Naoki Fukuda, Satoshi Endo, Yasuko Terada, Tomoko Abe, Akiko Hokura, Izumi Nakai

**Affiliations:** Japan Agency for Marine-Earth Science and Technology (JAMSTEC), 2-15 Natsushimacho, Yokosuka, Kanagawa 237-0068, Japan; Department of Applied Chemistry, Faculty of Science, Tokyo University of Science, 1-3 Kagurazaka, Shinjuku, Tokyo 162-8601, Japan; Fujita Co., 2025-1 Ono, Atsugi, Kanagawa 243-0125, Japan; Department of Applied Chemistry, Faculty of Science, Tokyo University of Science, 1-3 Kagurazaka, Shinjuku, Tokyo 162-8601, Japan; Department of Applied Chemistry, Faculty of Science, Tokyo University of Science, 1-3 Kagurazaka, Shinjuku, Tokyo 162-8601, Japan; Department of Applied Chemistry, Faculty of Science, Tokyo University of Science, 1-3 Kagurazaka, Shinjuku, Tokyo 162-8601, Japan; SPring-8, Japan Synchrotron Radiation Research Institute (JASRI), Sayo-cho, Hyogo 679-5198, Japan; RIKEN, 2-1 Hirosawa, Wako, Saitama 351-0198, Japan; Department of Applied Chemistry, School of Engineering, Tokyo Denki University, 5 Senju-Asahicho, Adachi, Tokyo 120-8551, Japan; Department of Applied Chemistry, Faculty of Science, Tokyo University of Science, 1-3 Kagurazaka, Shinjuku, Tokyo 162-8601, Japan

**Keywords:** synchrotron micro-XRF imaging, *Pteris vittata*, arsenic hyperaccumulator, frozen-hydrated section, root, As uptake pathway

## Abstract

We performed micro-X-ray fluorescence imaging of frozen-hydrated sections of a root of *Pteris vittata* for the first time, to the best of our knowledge, to reveal the mechanism of arsenic (As) uptake. The As distribution was successfully visualized in cross sections of different parts of the root, which showed that (i) the major pathway of As uptake changes from symplastic to apoplastic transport in the direction of root growth, and (ii) As and K have different mobilities around the stele before xylem loading, despite their similar distributions outside the stele in the cross sections. These data can reasonably explain As reduction, axially observed around the root tip in the direction of root growth and radially observed in the endodermis in the cross sections, as a consequence of the incorporation of As into the cells or symplast of the root. In addition, previous observations of As species in the midrib can be reconciled by ascribing a reduction capacity to the root cells, which implies that As reduction mechanisms at the cellular level may be an important control on the peculiar root-to-shoot transport of As in *P. vittata*.

## Graphical Abstract

**Graphical Abstract:**
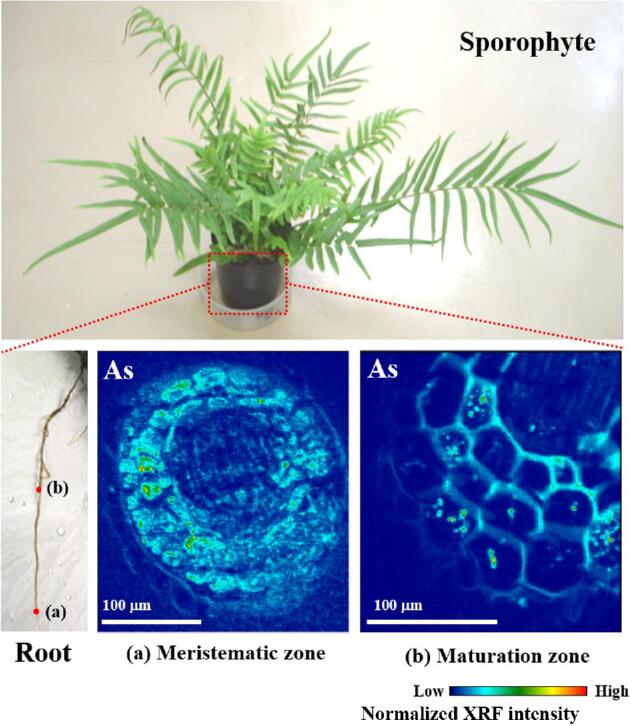
Synchrotron micro-X-ray fluorescence imaging combined with cryo-techniques visualized uptake pathways of arsenic (As) within a wet root of As hyperaccumulator, *Pteris vittata*.

## Introduction

*Pteris vittata* L. is the first terrestrial plant species identified to hyperaccumulate arsenic (As).^1^ This plant can accumulate As in its aboveground biomass up to several thousand mg kg^–1^ dry weight without suffering from high As toxicity, even when it is grown in the soils containing high levels of As in which the majority of plants cannot survive.^1,2^ Arsenic is naturally and anthropogenically occurring as a toxic contaminant of increasing global concern.^3^ This remarkable ability of *P. vittata* is ideal for removing As from contaminated soils and groundwater,^4^ which opened up phytoengineering of this peculiar fern and some of its relatives found afterward^5–7^ to an environmentally friendly remediation technology, called phytoremediation.^8^

Arsenic hyperaccumulation in *P. vittata* is also intriguing from a physiological standpoint because As is a highly toxic element to most biological systems.^9^ Arsenate [As(V)] acts as a phosphate (P) analog and interacts with the P metabolism, whereas arsenite [As(III)] reacts with the thiol (–SH) groups of enzymes and tissue proteins, leading to the disruption of cellular activity and death.^10,11^ These toxicities cause serious damage to most plants at relatively low As concentrations in plant tissues, which eventually limits As transport from roots to shoots in the plant body.^12,13^ Remarkably, *P. vittata* accumulates more than 90% of As in its fronds as more toxic As(III) form, even though As(V) is the dominant species in the oxic soils and water environments.^14–19^ Therefore, the reduction of As(V) to As(III) must occur in the plant body as a key part of the As hyperaccumulation mechanism. In fact, numerous studies have suggested that As reduction may contribute to various processes, including an efficient root uptake of As,^20^ its efficient transport from roots to shoots,^21,22^ and much-enhanced storage of and tolerance to As inside plant cells,^14,23^ all of which are unique features that distinguish As hyperaccumulators from non-hyperaccumulators.^13,24,25^

However, the mechanism of As reduction in *P. vittata* is still unknown, largely because detailed information about the As reduction site in the plant body is lacking. Many observations suggest that As is transported as As(V) from the roots to the fronds, and is reduced to As(III) mainly in the fronds.^26,27^ Some evidence implies that this As(III) may be stored in vacuoles to sequester its toxicity in the cells.^14^ On the other hand, other evidence suggests that the roots are the main As reduction site because (i) the major As species in the xylem sap of *P. vittata* is As(III), regardless of the As species present in the growth medium,^22,28^ and (ii) the activity of arsenate reductase was detected only in the roots by molecular biology.^29^ Further alternate evidence suggests that As could be reduced, enzymatically or non-enzymatically, in the gametophyte of *P. vittata* as a constitutive consequence of cellular metabolisms not associated with the specific functions of the sporophyte structures such as roots, stems, and fronds.^30–32^ These individual observations of As(III) in various tissues and life stages of the plant body indicate that further understanding of As reduction mechanisms in relation to physiological/biochemical activities of *P. vittata* is required.

**Fig. 1:**
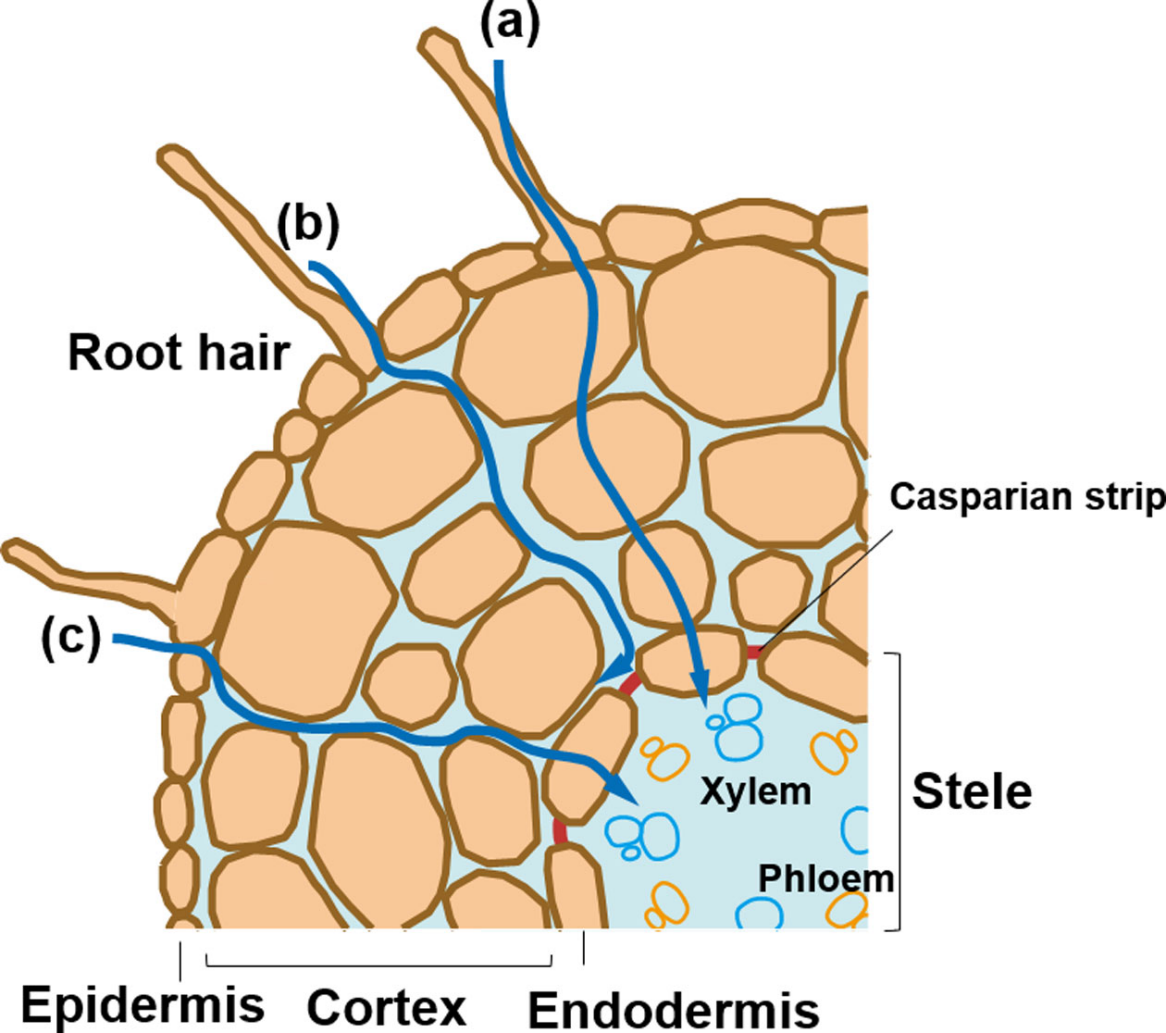
Typical inner structure of a root and ion transports. Curbed blue arrows represent ion transports via (a) symplast, which comprises spaces on the inner side of the plasma membrane that are connected by plasmodesmata, (b) apoplast, which comprises spaces on the outer side of the plasma membrane formed by the continuum of adjacent cell walls, and (c) switching from apoplast to symplast around the endodermis, where the Casparian strip acts as an apoplastic barrier of the ion transport.

In this study, we focused on As behavior in roots, which are increasingly recognized as the first As reduction site.^29,33^ Previous studies, mostly bulk experiments, have strongly indicated that As reduction occurs in the roots.^13,22,34^ However, further microscopic observations of As(III) by several previous studies have not necessarily been interpreted in the context of As uptake mechanisms,^33,35,36^ because the As transport pathways in roots are unknown. In those studies, several spot analyses by micro-X-ray absorption near edge structure (XANES) of root cross sections have slightly detected As(III) in the endodermis and vascular bundle, suggesting that As reduction may occur in the endodermis during its radial transport.^33,35^ On the other hand, in axial XANES analysis in the direction of root growth, As(III) has been detected only around the root tip, indicating that the As uptake pathway could be different depending on the region in a root.^36^ For a comprehensive understanding of these individual XANES evidences, it is necessary to reveal the As distribution in microstructures of a root, including the epidermis, cortex, endodermis, and stele, each of which has a different role in the root functions (Fig. 1).

A major impediment to performing high-spatial-resolution analyses of roots is the difficulty of handling in the air, because root is highly hydrated tissue without the cuticle layer on the surface, making it particularly soft and susceptible to drying. Previous microanalyses of the roots of *P. vittata* employed a freeze-drying technique,^33,35,36^ where the samples are first frozen and then dried for storage at room temperature. This technique not only allows easy handling of the samples in the air but also causes artifacts as a result of dehydration, which makes it difficult to collect the data at the tissue or cellular level as close to the living state.^37–39^ Here, we applied cryo-techniques to both sectioning and measurement to perform synchrotron micro-X-ray fluorescence (micro-XRF) analyses of “frozen-hydrated” sections of the roots of *P. vittata*. This method simply fixes the water molecules by quick freezing to preserve cell structures and elemental distribution;^40^ therefore, the obtained data should be the closest to the living state of the plants.^37–39^ While the number of the attempts to analyze wet roots by using non-destructive advantage of X-ray technique is increasing for other plant species,^41^ this is, to the best of our knowledge, the first analysis of frozen-hydrated sections of the root of *P. vittata* to clarify As behavior at the cellular level in a root.

The purpose of this study is to reveal the As distribution along the root structures, which is an important piece to connect the As uptake and reduction mechanisms in the roots of *P. vittata*. We performed micro-XRF imaging of frozen-hydrated sections of the different parts of the same root, and the findings at the tissue/cellular levels are discussed in relation to the As species observed in previous XANES studies. This study could contribute significantly to unravel the roles of the root of *P. vittata* in As uptake and transport, which is at the forefront of As hyperaccumulation in the plant body.

## Experimental

### Plant culture and arsenic treatment

*Pteris vittata* were cultivated for 6 months from spores germinated on potting soils, consisting of peat moss and vermiculite, soaked with 1/10 strength Murashige and Skoog (MS) medium (Nihon Pharmaceutical Co. Ltd). The seedlings were then transferred to a hydroponic culture of 1/10 MS liquid medium with no arsenic and were cultured for 2 weeks. After confirming the growth of fresh roots, the nutrient solution was changed to a 10 mg L^–1^ As solution made by dissolving KH_2_AsO_4_ (Kanto Chemical Co. Ltd) into the medium. The plants were exposed to the As solution for 3 days. It seemed that the root did not show significant change at a glance after 3 days independent of the As exposure. All the cultivation was conducted under the conditions of 14 h of light at an intensity of 228 μmol/(m^2^ s), day/night temperatures of 27.5°C/22.5°C, and a relative humidity of 70–80%. Cultivation under similar conditions previously led to As concentrations in fronds and roots of ∼140 and 120 mg/kg dry weight, respectively.^42^

### Preparation of frozen-hydrated sections of a root

To prepare frozen-hydrated sections of roots from As-treated plants, the roots were first rinsed with deionized water. Then, a main root was divided into segments of ∼5 mm above the root tip by razor blade. These segments were quickly immersed in optical cutting temperature compound (SAKURA Finetek, Tokyo, Japan), and were immediately frozen on an aluminum block that had been pre-cooled to the optimal cutting temperature of −20°C. These procedures were finished within a few minutes. The solid blocks thus obtained were cut into 20-μm-thick cross sections using a cryomicrotome (LEICA CM-3050S). Sections were prepared from different parts of the same root, including meristematic, elongation, and maturation zones, depending on their different developments. The prepared cross sections were mounted with double-faced tape on an acryl plate with a 3-mm-diameter hole in the center in the cryomicrotome. Then, the acryl plates were set in a pre-cooled sample holder for μ-XRF analysis and stored as frozen at −80°C in a freezer until analysis.

### Synchrotron μ-XRF imaging under cryogenic condition

Two-dimensional μ-XRF imaging was performed at BL37XU in SPring-8 (Hyogo, Japan). The beam from the undulator was monochromatized at 12.8 keV by a Si(111) double crystal monochromator. The incident X-ray was focused by a K-B mirror system to the size of 1.5 μm (vertical, V) × 2.3 μm (horizontal, H) on the sample surface. Samples were mounted on an XY stage placed at 10° to the incident X-ray beam, and were spatially scanned in steps of 1.0 μm (V) × 1.0 μm (H) by using stepping motors. The intensity of the incident X-ray (*I*_0_) was monitored by using an ionization chamber filled with the air. Fluorescence X-rays from the samples were collected by silicon drift detector placed at 90° to the incident X-ray beam, and integrated for 0.1 s at each point. The integrated peak intensity of the XRF line of each element per pixel was normalized by *I*_0_. Elemental maps of the measurement areas were displayed on a 256-gradation color scale, with red denoting the highest intensities and blue the lowest. The distances between the samples and the detector were fixed so that individual measurements of XRF intensity from the elements of interest in each cross section of the sample could be compared.

To keep the samples frozen during the several hours of measurements, the samples were exposed to a cryogenic N_2_ gas stream (Cryojet XL, Oxford Instruments, UK). In addition, a Mylar film^®^ (SPC Science, Champlain, NY, USA) was attached behind the acryl plate to block the flow of moist air from behind the samples. This procedure is also important to prevent the frost occurring around the samples, which would decrease the objective XRF signals and increase background noise due to X-ray scattering.

## Results and discussion

### Technical significance of μ-XRF imaging of frozen-hydrated sections of a root of *P. vittata*

**Fig. 2:**
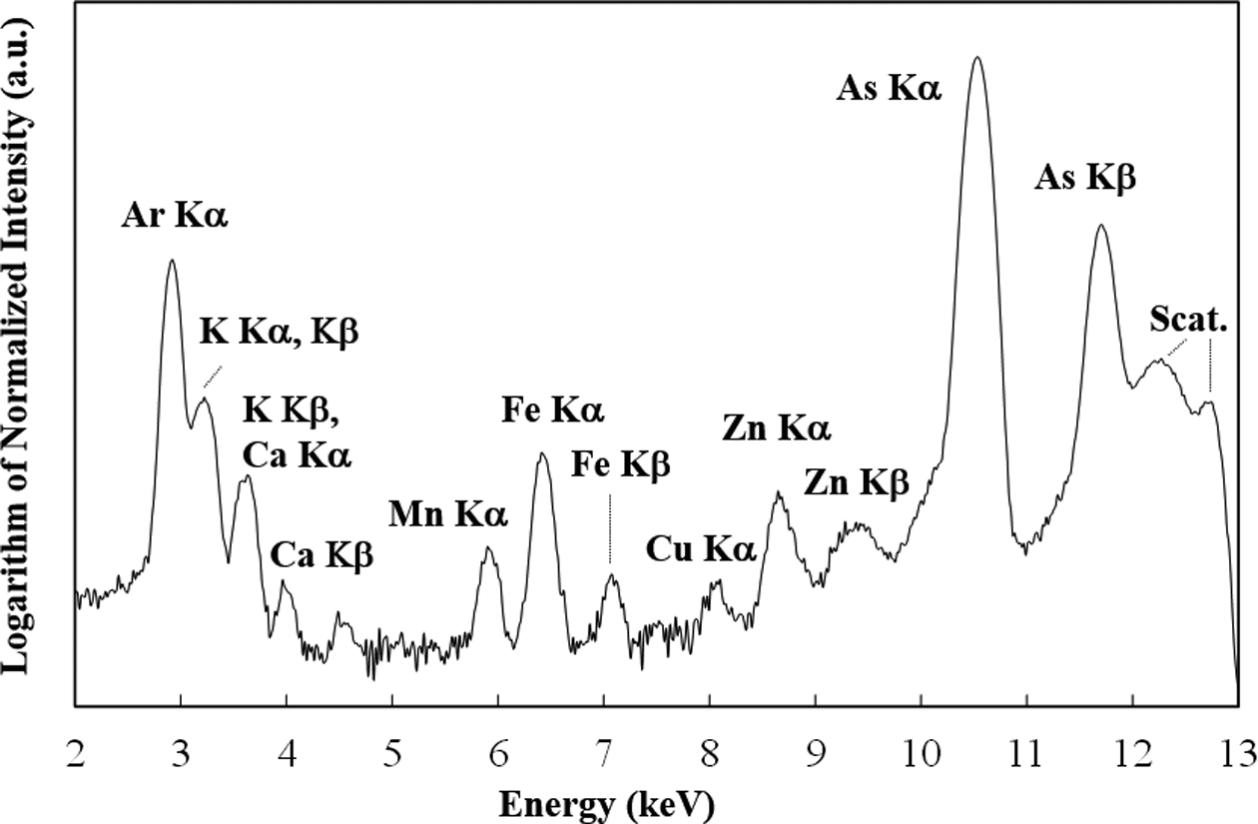
An example of an XRF spectrum of a cross section of a root of *P. vittata* under a cryogenic N_2_ gas stream. Excitation energy: 12.8 keV, integration time: 300 s.

**Fig. 3:**
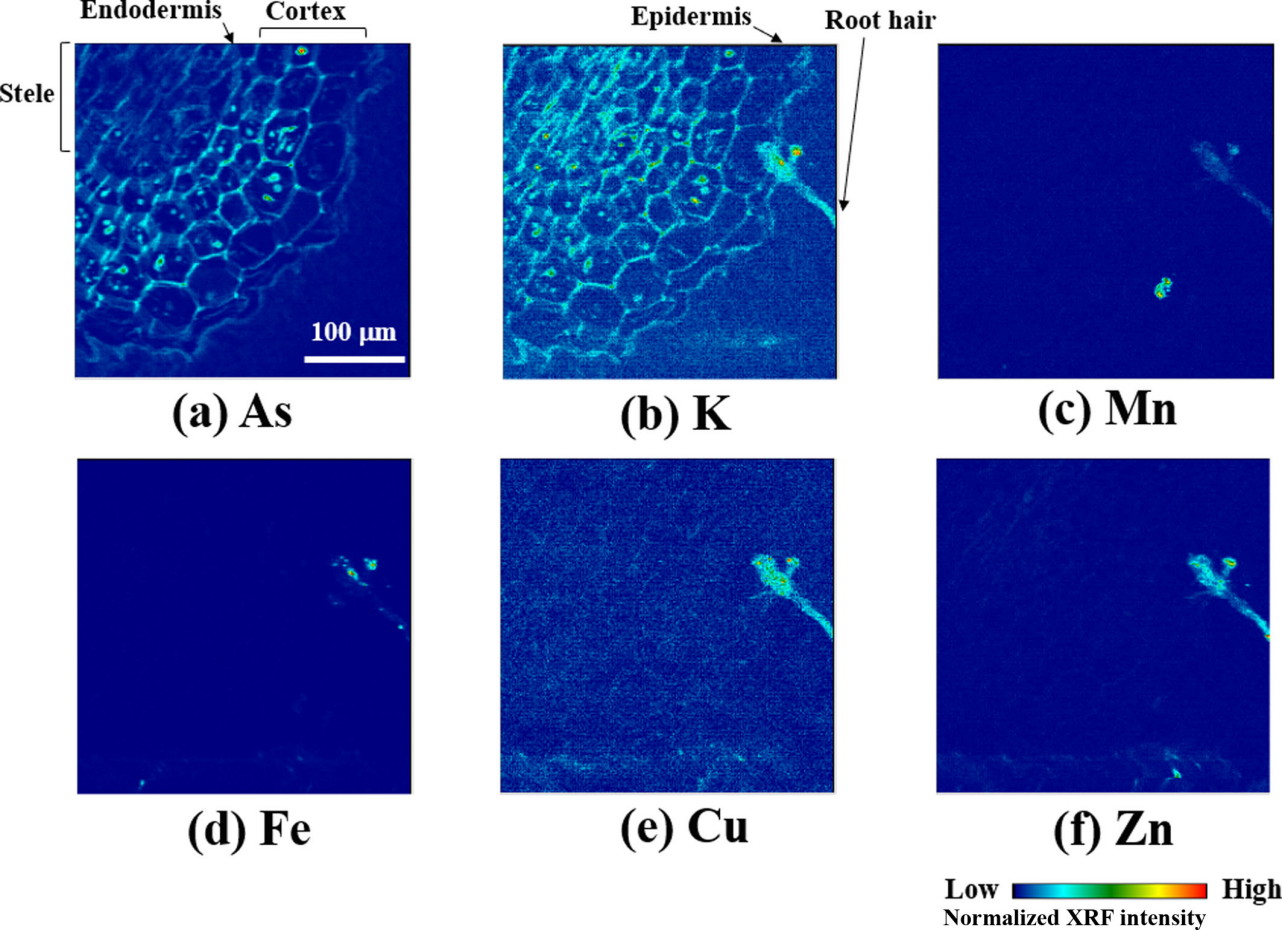
Micro-XRF imaging of a cross section of the maturation zone of a root of *P. vittata*. Excitation energy: 12.8 keV. Beam size: 1.1 (V) × 1.3 (H) μm^2^, step size: 1.0 (V) × 1.0 (H) μm^2^, integration time: 0.2 s/point.

Figure 2 shows as an example of the micro-XRF spectrum from a cross section of maturation zone of a root (∼2 cm from the tip) measured in the cryogenic N_2_ gas stream. Sharp peaks of As K lines were observed at all analyzed spots with slight detection of elements such as K, Mn, Fe, Cu, and Zn in some cases. The weakness of the X-ray signals, especially those of the lighter essential elements, is likely a consequence of measuring the samples in their frozen-hydrated state. Nevertheless, the detected elements were collected to obtain the elemental 2D maps of the cross sections of the root as shown in Fig. 3.

The maps show As and K spread over the whole region of the cross section (Fig. 3a and b), whereas Mn, Fe, Cu, and Zn are localized along the accessory structure like a root hair (Fig. 3c–f). Arsenic is found mainly along cell walls, which delineates the typical structures of a matured root, including the epidermis, cortex, endodermis, and stele (see Fig. 1), and it also occurs as speckles inside the cells of the cortex and endodermis (Fig. 3a). Low As signals in the center of the root, relative to the cell layers of the cortex and endodermis, highlight the shape of the stele with faint shapes of vascular bundles (Fig. 3a). Similarly, K is found along the cell walls and as speckles inside the cells, but it clearly contrasts to As in the stele, where signal of K does not weaken within the stele (Fig. 3b). Potassium is also distributed along the root hair (Fig. 3b) as is the case for the other essential elements such as Mn, Fe, Cu, and Zn (Fig. 3c–f), but is not for As (Fig. 3a).

These data demonstrate our successful application of the frozen-hydrated technique to micro-XRF analysis of the root of *P. vittata*, because the data apparently preserve typical inner structures of a root, including symmetric shapes of cells and elemental localization inside the cell, with minimal perturbation at the tissue/cellular level (Fig. 1), and are also showing systematic differences among the cross sections in the development of the root structures (as described in the section “Pathways of As uptake in the structure of a root”). In the freeze-drying technique, the most common technique used by previous studies, the dehydration step can lead to various artifacts, such as shrinkage of cell structures, the presence of empty cells, and the redistribution of elements,^37,39^ some of which are actually observed in the previous microanalyses of the roots of *P. vittata.*^33,35^ Therefore, we can recognize that the elemental maps obtained here clearly show one of the benefits of minimal handling of water, which is the main issue in sample preparation.^38^

The high spatial resolution of the elemental maps is originated partly from cryosectioning, which is another benefit in the frozen-hydrated technique.^38^ In principle, spatial resolution in X-ray analysis is controlled by not only the beam size but also the sample thickness, because information from the probed area is averaged over the penetration depth of X-rays. Here, the frozen-hydrated technique can provide thinner sections compared with the freeze-drying technique, which practically requires thicker sections to avoid shrinkage by dehydration.^37^ This procedural advantage actually helped us to obtain clear elemental maps that could be used to discuss the relationship with physiological processes in the root (Fig. 3, see the following sections). In particular, we could observe elemental localization inside of cells (Fig. 3a and b), which is remarkable even in comparison with our previous micro-XRF analyses of the aboveground parts of the plant bodies obtained by the same technique.^43,44^ This is partly because the symmetric shape of roots and their high water content make it easier to prepare thin sections, compared with more complex aboveground tissues in which different density materials alternate in the structures. Thus, our results suggest that the root could be one of the ideal samples for the application of the frozen-hydrated technique, although handling of it is difficult in the air.

On the other hand, there are limitations of the frozen-hydrated technique that occurs as trade-off of the two benefits above: the retention of water and sample thinness.^39^ First, both the presence of water in the samples and the sample thinness reduce the intensity of XRF signals. Therefore, the sensitivity to essential elements, which are mostly light elements, tends to be low under cryogenic conditions, as inferred from Fig. 2. Second, morphological observation of the same samples after they have been analyzed by micro-XRF is difficult because, once samples are frozen, their structures easily collapse upon melting at room temperature. As a consequence, our measurements consistently achieved high sensitivity only for As detection, which is a heavy element accumulated in the plant tissues, and we could not perform detailed morphological observations of the same cross sections (see the section “Pathways of As uptake in the structure of a root”).

Nevertheless, this first application of the frozen-hydrated technique in micro-XRF analysis successfully visualized As localization in cross sections of a root of *P. vittata*. The data are significantly clear at the tissue/cellular levels and the closest to the living state of the plant. These advantages indicate the robustness of this technique for revealing the pathways of As uptake in the roots of *P. vittata*, as discussed in the following sections.

### Pathways of As uptake in the structure of a root

**Fig. 4:**
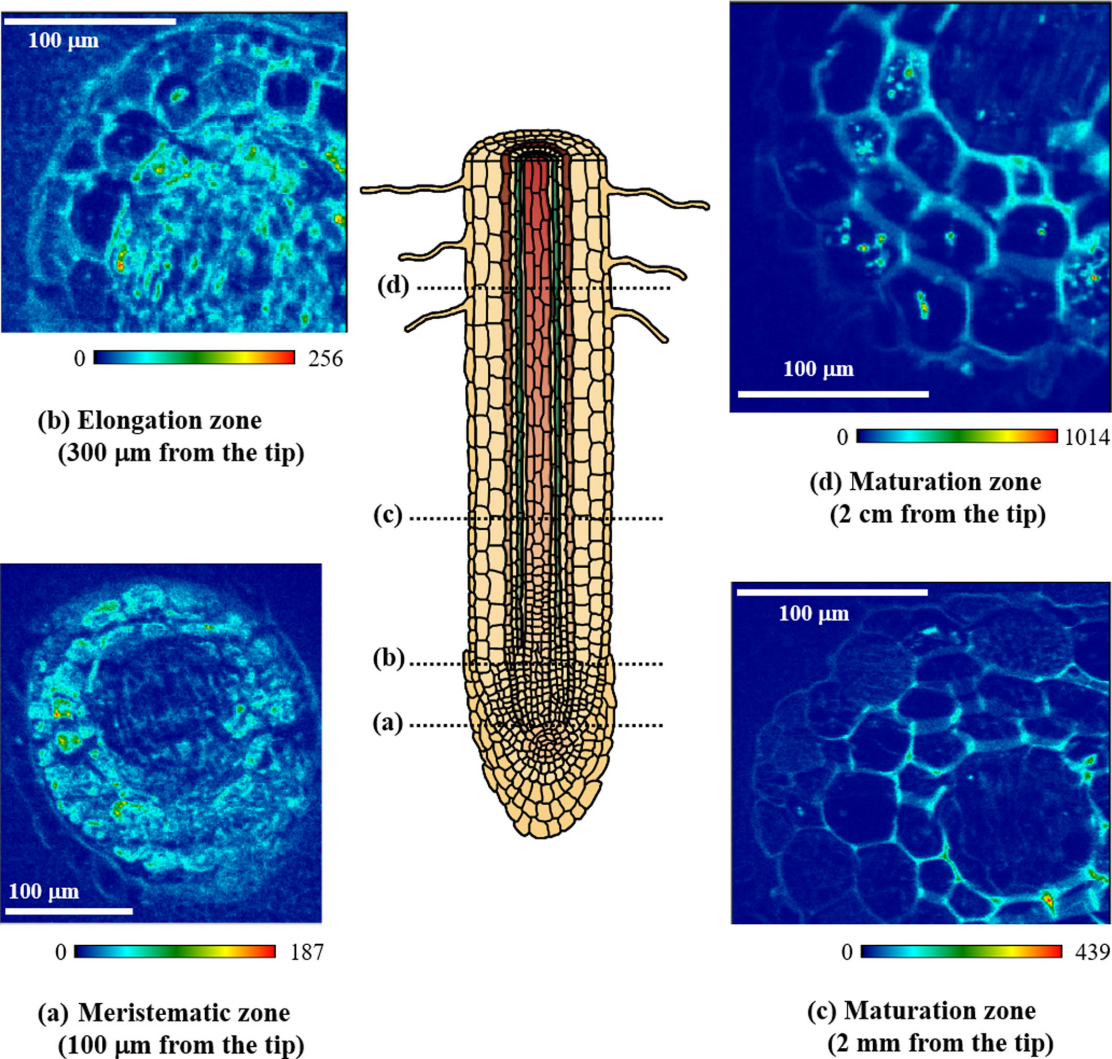
Arsenic distributions in frozen-hydrated sections of different parts of a root of *P. vittata* obtained by micro-XRF imaging. Excitation energy: 12.8 keV. Beam size: 1.5 (V) × 2.3 (H) μm^2^, step size: 1.0 (V) × 1.0 (H) μm^2^, integration time: 0.1 s/point. The colored bars show the XRF intensity of as obtained at a fixed distance from the detector.

Figure 4 shows As distributions on cross sections from different parts of a root. In the meristematic zone of 100 μm from the tip (Fig. 4a), As is distributed over the whole area, including the inside of the tiny cells, with the relatively low signal around the center of the root. In the elongation zone of 300 μm from the tip (Fig. 4b), the As is distributed in the central region and also localized along the developing cell walls of the outer layer of the root. In the maturation zone of the root of 2 mm from the tip, where the root is whitish (Fig. 4c), the development of individual cells is highlighted by the localization of As along cell walls, which enables us to recognize typical structures of the root tissues, including the stele. In the maturation zone of 2 cm from the tip, where the root is blackish (Fig. 4d), As is mostly localized along cell walls and is also found as speckles inside of the cells, as in Fig. 3a. We revealed that the As distributions within a root depend on its developmental stage.

These differences in As distributions among the cross sections indicate that there are several pathways for radial transport of the As uptake in the root. Near the tip of the root (Fig. 4a and b), homogeneous distribution of As found in the tiny cells indicates that the As is transported mainly via the symplast, which comprises spaces on the inner side of the plasma membrane that are connected by plasmodesmata.^45^ In contrast, in the regions farther from the tip of the root (Fig. 4c and d), the localization of As along the cell walls indicates its transport via the apoplast, which comprises spaces on the outer side of the plasma membrane formed by the continuum of adjacent cell walls.^45^ These two pathways of As transport apparently change in their relative importance in the direction of root growth.

This transition between As uptake pathways also seems to correlate with changes of XRF intensity of As with root growth. The overall intensity of As on these maps, which were all collected at the same fixed distance from the detector, systematically increases from the tip to the base of the root: this is approximately recognized by maximum As intensity increased in the order of 187 (Fig. 4a), 246 (Fig. 4b), 439 (Fig. 4c), and 1014 cps (Fig. 4d). Although the XRF intensity here should be distinguished from the elemental concentration, this increase agrees with the findings of Kashiwabara *et al.* (2006), who obtained axial profiles of XRF intensities by synchrotron X-ray analysis that showed a systematic increase in As from the tip to the base of the root.^36^ In general, different parts of a root could show different degree of growth rates, maturation, and requirements of nutrients depending on their own developmental stages, and younger parts with higher metabolic activities are believed to be more active in nutrient uptake.^45^ These physiological situations could be also observed in the As distributions, where the region around the tip (Fig. 4a and b) may be more active in cell divisions. On the other hand, in the case of As, the uptake by the root may be more active toward the base where structures are already matured.

Comparison with the K distribution gives further insights into the As uptake mechanism, although low XRF intensity of K provided only limited maps shown in Fig. 3b. In the most regions, K is localized along the cell walls like As (Fig. 3a and b). This similarity between As and K indicates that K serves as the dominant counterbalancing cation to As in the root, which is a general role of K in the nutrient uptake.^46^ In particular, in the case of *P. vittata*, the effect of K in plant growth has long been implied in many previous studies, where As uptake results in the enhancement of the biomass of the fronds with increased K concentration.^14,47,48^ This close relationship between As and K observed in plant growth could be partly visualized in this study as their similar behavior in the radial transport of the nutrient uptake.

On the other hand, there is a clear contrast between As and K in the stele: As is absent but K is present (Fig. 3a and b). This contrast is also suggestive to the mechanism of As uptake because the stele is a key structure at the interface between the radial transport of nutrients in the root and their root-to-shoot transport.^49^ In general, the ions are transported radially via both the apoplastic and symplastic pathways, but their transport across the endodermis is forced via the symplast because the Casparian strip in the endodermis acts as an apoplastic barrier that prevents ions in the apoplast from entering into the stele (Fig. 1).^50^ Therefore, the contrasting distribution of these two elements in the stele (Fig. 3a and b) indicates their different mobility for entering into the stele. It may imply the existence of potential biological mechanisms specific to the endodermis of *P. vittata* that results in the peculiar root-to-shoot transport of As that does not occur in normal plants.

Here, we emphasize that (i) distinguishing two major pathways of As uptake and (ii) the comparison between As and K both represent significant progresses achieved by the frozen-hydrated technique because they are closely related to the presence of water. These findings could provide further insights into the tolerance to As toxicity in *P. vittata* when it is compared with behavior of As and/or essential elements in the root of other plants sensitive to the As toxicity. In addition, we should note that the observations of speckles of As inside the cells in the maturation zone (Fig. 3a and Fig. 4d) represent another progress in As localization revealed at the cellular scale in the root of *P. vittata*. At present, these speckles have many possibilities in the context of the root activity such as (i) vesicles in endocytosis as another pathway of uptake into cells,^45,51^ (ii) storage in the vacuoles of cells,^14,52^ or (iii) cytoplasmic precipitations as a tolerance mechanism as has been shown in the plants resistant to other metals.^53,54^ In any case, these speckles, visualized here for the first time by frozen-hydrated technique, imply the existence of cellular processes for As accumulation in the root of *P. vittata*, but further subcellular-scale analysis is required for their identification.

### Relationship between the As uptake and reduction mechanisms in a root

We previously performed XANES analysis of a freeze-dried root in an axial direction, and found As(III) around the tip and As(V) around the base.^36^ Their proportions gradually changed from the tip to the base; this suggested a different mechanism of As uptake depending on the developmental stage, even in the same root.^36^ This axial transition of the major As species from As(III) to As(V) can be explained by our finding that the As transport pathways transition from symplastic to apoplastic with root growth (Fig. 4). In particular, As(V) is a reasonable species of As in the apoplast because the apoplastic space is outside of the root cells. Similarly, the presence of As(III) in the symplast can be reasonably inferred because As reduction is expected to be a consequence of biological processes on/inside the plasma membrane of the root cells. Therefore, As reduction in the root seems to be accompanied by the incorporation of As inside cells.

This relationship between As species and spaces inside or outside of root cells can provide a framework to understand a series of previous observations. First, previous micro-XANES results for cross sections of freeze-dried root^33^ suggested that the endodermis may be the structure in which As(III) reduction occurs, because the several-spot analyses found As as As(V) from the epidermis to the cortex and the proportion of As(III) increased from the endodermis to the vascular bundle. This hypothesis is interesting because the endodermis controls xylem loading; thus, As reduction might be related at the tissue level to the ability of *P. vittata* to efficiently transport As from the root to the shoot.^26,13^ On the other hand, if the As reduction observed in the endodermis is related to the switch from the apoplastic to the symplastic pathways around the stele (Fig. 1),^50^ the same mechanism could also work at the cellular level via the plasma membrane of the root cells. In fact, reduced As has been observed around the root tip^36^ where the stele has not developed and As is present in the symplast (this study: Fig. 4a). Therefore, we suggest that the As reduction should be recognized as a cellular process, a consequence of its incorporation into the root cells or the symplast, which can provide a comprehensive explanation for both previous axial and radial XANES observations of As species in the root of *P. vittata*.

Second, this As reduction should also be discussed in relation to root-to-shoot transport, because many previous observations still found significant amounts of As(V) in the midrib of *P. vittata*.^26,27,55^ If As reduction could be accompanied by passage through the symplast of the root before the xylem loading (Fig. 1), then all the As species in root-to-shoot transport should be As(III). In this context, one idea to explain this As(V) observation in the midrib may be the existence of potential mechanism of As(III) oxidation during xylem transport. On the other hand, other studies have found As(III) to be the main As species in the xylem sap regardless of whether the plants were exposed to As(V) or As(III).^22,28^ We hypothesize that this contradiction can be reconciled by ascribing “a capacity of As reduction” to the root cells. That is, most As could be reduced in the symplast of the root when the As concentration in growth media is low, but at higher As concentrations, some As might not be reduced, because the reduction capacity of the root cells is exceeded. This possibility is supported by the reported trend that higher As(V) concentrations in growth medium tend to result in an increase in the proportion of As(V) in the xylem transport,^56^ although most studies were performed at one level or within a limited range of As concentration.

This hypothesis drives further interest in the relationship between cellular mechanisms in the root and the whole system of the As hyperaccumulation in *P. vittata*. Many studies that have investigated the different behaviors of As(V) and As(III) in the plant body of *P. vittata*^13,20,56,57^ have suggested that the rate of As(III) translocation to the aboveground part is higher than that of As(V).^58^ In this context, the capacity of root cells to reduce As, if it exists, can be an important control on the translocation of As to the shoot in *P. vittata*. Interestingly, an As reduction mechanism in the cells could also control the As distribution in the plant body for the case of gametophyte systems, where different ferns show different As distributions in their cellular sheets depending on whether the reduced As(III) species is coordinated by O or by S.^31,59^ Therefore, our study implies that understanding the As reduction mechanism at the cellular level in the roots is important for understanding the unique physiology that leads to As hyperaccumulation in *P. vittata*.

## Conclusion

The application of the frozen-hydrated technique in micro-XRF imaging of the root of *P. vittata* was successful. We revealed that the major pathway of the As uptake changes from symplastic to apoplastic transport in the direction of root growth, and also found the different mobilities of As and K around the stele in the radial transport. The findings here can reasonably explain previous XANES observations of As species in the root by assuming As reduction as a consequence of its incorporation into the root cells or symplast, and they also imply that the As reduction capacity of root cells may be an important control on the peculiar As transport from the root to the shoot in *P. vittata*.

## Data Availability

The data underlying this article will be shared on reasonable request to the corresponding author.

## References

[1] Ma L. Q. , Komar K. M., Tu C., Zhang W., Cai Y., Kennelley E. D., A fern that hyperaccumulates arsenic, Nature, 2001, 409 (6820), 579. 11214308 10.1038/35054664

[2] Tu S. , Ma L. Q., Interactive effects of pH, arsenic and phosphorus on uptake of As and P and growth of the arsenic hyperaccumulator *Pteris vittata* L. under hydroponic conditions, Environ. Exp. Bot., 2003, 50 (3), 243–251.

[3] Smedley P. L. , Kinniburgh D. G., A review of the source, behaviour and distribution of arsenic in natural waters, Appl. Geochem., 2002, 17 (5), 517–568.

[4] Raskin I. , Ensley B. D., Phytoremediation of Toxic Metals, Wiley, New York, 2000.

[5] Zhao F. , Dunham S., McGrath S., Arsenic hyperaccumulation by different fern species, New Phytol., 2002, 156 (1), 27–31.

[6] Meharg A. A. , Variation in arsenic accumulation–hyperaccumulation in ferns and their allies: rapid report, New Phytol., 2003, 157 (1), 25–31. 33873706 10.1046/j.1469-8137.2003.00541.x

[7] Li J. T. , Gurajala H. K., Wu L. H., van der Ent A., Qiu R. L., Baker A. J. M., Tang Y. T., Yang X. E., Shu W. S., Hyperaccumulator plants from China: a synthesis of the current state of knowledge, Environ. Sci. Technol., 2018, 52 (21), 11980–11994. 30272967 10.1021/acs.est.8b01060

[8] Krämer U. , Phytoremediation: novel approaches to cleaning up polluted soils, Curr. Opin. Biotechnol., 2005, 16 (2), 133–141. 15831377 10.1016/j.copbio.2005.02.006

[9] M. G. , Ord, L. A., Stockton A contribution to chemical defense in World War II. Trends Biochem. Sci. 2000, 25 (5), 253–256. 10782099 10.1016/s0968-0004(00)01578-4

[10] Vahter M. , Mechanisms of arsenic biotransformation, Toxicology, 2002, 181–182, 211–217. 10.1016/s0300-483x(02)00285-8 12505313

[11] Meharg A. A. , Hartley-Whitaker J., Arsenic uptake and metabolism in arsenic resistant and nonresistant plant species, New Phytol., 2002, 154 (1), 29–43.

[12] Tu S. , Ma L. Q., Comparison of arsenic and phosphate uptake and distribution in arsenic hyperaccumulating and nonhyperaccumulating fern, J. Plant Nutr., 2005, 27 (7), 1227–1242.

[13] Singh N. , Ma L. Q., Arsenic speciation, and arsenic and phosphate distribution in arsenic hyperaccumulator *Pteris vittata* L. and non-hyperaccumulator *Pteris ensiformis* L, Environ. Pollut., 2006, 141 (2), 238–246. 16257102 10.1016/j.envpol.2005.08.050

[14] Lombi E. , Zhao F. J., Fuhrmann M., Ma L. Q., McGrath S. P., Arsenic distribution and speciation in the fronds of the hyperaccumulator *Pteris vittata*, New Phytol., 2002, 156 (2), 195–203. 33873285 10.1046/j.1469-8137.2002.00512.x

[15] Wang J. , Zhao F. J., Meharg A. A., Raab A., Feldmann J., McGrath S. P., Mechanisms of arsenic hyperaccumulation in *Pteris vittata*. Uptake kinetics, interactions with phosphate, and arsenic speciation, Plant Physiol., 2002, 130 (3), 1552–1561. 12428020 10.1104/pp.008185 PMC166674

[16] Tu C. , Ma L. Q., Zhang W., Cai Y., Harris W. G., Arsenic species and leachability in the fronds of the hyperaccumulator Chinese brake (*Pteris vittata* L.), Environ. Pollut., 2003, 124 (2), 223–230. 12713922 10.1016/s0269-7491(02)00470-0

[17] Chen R. , Smith B. W., Winefordner J. D., Tu M. S., Kertulis G., Ma L. Q., Arsenic speciation in Chinese brake fern by ion-pair high-performance liquid chromatography–inductively coupled plasma mass spectroscopy, Anal. Chim. Acta, 2004, 504 (2), 199–207.

[18] Luongo T. , Ma L., Characteristics of arsenic accumulation by *Pteris* and non-*Pteris* ferns, Plant Soil, 2005, 277 (1–2), 117.

[19] Webb S. M. , Gaillard J.-F., Ma L. Q., Tu C., XAS speciation of arsenic in a hyper-accumulating fern, Environ. Sci. Technol., 2003, 37, 754–760. 12636275 10.1021/es0258475

[20] Wang X. , Ma L. Q., Rathinasabapathi B., Cai Y., Liu Y. G., Zeng G. M., Mechanisms of efficient arsenite uptake by arsenic hyperaccumulator *Pteris vittata*, Environ. Sci. Technol., 2011, 45 (22), 9719–9725. 22029254 10.1021/es2018048

[21] Poynton C. Y. , Huang J. W., Blaylock M. J., Kochian L. V., Elless M. P., Mechanisms of arsenic hyperaccumulation in *Pteris* species: root As influx and translocation, Planta, 2004, 219 (6), 1080–1088. 15221388 10.1007/s00425-004-1304-8

[22] Su Y. H. , McGrath S. P., Zhu Y. G., Zhao F. J., Highly efficient xylem transport of arsenite in the arsenic hyperaccumulator *Pteris vittata*, New Phytol., 2008, 180 (2), 434–441. 18662326 10.1111/j.1469-8137.2008.02584.x

[23] Indriolo E. , Na G., Ellis D., Salt D. E., Banks J. A., A vacuolar arsenite transporter necessary for arsenic tolerance in the arsenic hyperaccumulating fern *Pteris vittata* is missing in flowering plants, Plant Cell, 2010, 22 (6), 2045–2057. 20530755 10.1105/tpc.109.069773 PMC2910956

[24] Fayiga A. , Ma L., Santos J., Rathinasabapathi B., Stamps B., Littell R., Effects of arsenic species and concentrations on arsenic accumulation by different fern species in a hydroponic system, Int. J. Phytoremediation, 2005, 7 (3), 231–240. 16285413 10.1080/16226510500215720

[25] Fayiga A. , Ma L., Arsenic uptake by two hyperaccumulator ferns from four arsenic contaminated soils, Water Air Soil Pollut., 2005, 168 (1–4), 71–89.

[26] Kertulis G. , Ma L., MacDonald G., Chen R., Winefordner J., Cai Y., Arsenic speciation and transport in *Pteris vittata* L. and the effects on phosphorus in the xylem sap, Environ. Exp. Bot., 2005, 54 (3), 239–247.

[27] Pickering I. J. , Gumaelius L., Harris H. H., Prince R. C., Hirsch G., Banks J. A., Salt D. E., George G. N., Localizing the biochemical transformations of arsenate in a hyperaccumulating fern, Environ. Sci. Technol., 2006, 40, 5010–5014. 16955900 10.1021/es052559a

[28] Huang Z.-C. , Chen T.-B., Lei M., Hu T.-D., Direct determination of arsenic species in arsenic hyperaccumulator *Pteris vittata* by EXAFS, Acta Bot. Sinica, 2004, 46, 46–50.

[29] Duan G.-L. , Zhu Y.-G., Tong Y.-P., Cai C., Kneer R., Characterization of arsenate reductase in the extract of roots and fronds of Chinese brake fern, an arsenic hyperaccumulator, Plant Physiol., 2005, 138 (1), 461–469. 15834011 10.1104/pp.104.057422 PMC1104199

[30] Ellis D. R. , Gumaelius L., Indriolo E., Pickering I. J., Banks J. A., Salt D. E., A novel arsenate reductase from the arsenic hyperaccumulating fern *Pteris vittata*, Plant Physiol., 2006, 141 (4), 1544–1554. 16766666 10.1104/pp.106.084079 PMC1533930

[31] Kashiwabara T. , Mitsuo S., Hokura A., Kitajima N., Abe T., Nakai I., *In vivo* micro X-ray analysis utilizing synchrotron radiation of the gametophytes of three arsenic accumulating ferns, *Pteris vittata* L., *Pteris cretica* L. and *Athyrium yokoscense*, in different growth stages, Metallomics, 2010, 2 (4), 261–270. 21069168 10.1039/b922866g

[32] Cesaro P. , Cattaneo C., Bona E., Berta G., Cavaletto M., The arsenic hyperaccumulating *Pteris vittata* expresses two arsenate reductases, Sci. Rep., 2015, 5 (1), 14525. 26412036 10.1038/srep14525 PMC4585942

[33] Lei M. , Wan X. M., Huang Z. C., Chen T. B., Li X. W., Liu Y. R., First evidence on different transportation modes of arsenic and phosphorus in arsenic hyperaccumulator *Pteris vittata*, Environ. Pollut., 2012, 161, 1–7. 22230060 10.1016/j.envpol.2011.09.017

[34] W. , Ma L. Q. Arsenic speciation and distribution in an arsenic hyperaccumulating plant. Sci. Total Environ. 2002, 300 (1–3), 167–177. 12685480 10.1016/s0048-9697(02)00165-1

[35] Wan X. , Lei M., Chen T., Ma J., Micro-distribution of arsenic species in tissues of hyperaccumulator *Pteris vittata* L, Chemosphere, 2017, 166, 389–399. 27705826 10.1016/j.chemosphere.2016.09.115

[36] Kashiwabara T. , Hokura A., Kitajima N., Onuma R., Saito H., Abe T., Nakai I., Distribution and oxidation state of arsenic in root of arsenic-hyperaccumulator fern, *Pteris vittata* L., by using synchrotron radiation X-ray fluorescence analysis, Bunseki Kagaku, 2006, 55 (10), 743–748.

[37] Sarret G. , Smits E. P., Michel H. C., Isaure M., Zhao F., Tappero R., Advances in Agronomy, Vol. 119, Elsevier, 2013, 1–82.

[38] Castillo-Michel H. A. , Larue C., del Real A. E. P., Cotte M., Sarret G., Practical review on the use of synchrotron based micro-and nano-X-ray fluorescence mapping and X-ray absorption spectroscopy to investigate the interactions between plants and engineered nanomaterials, Plant Physiol. Biochem., 2017, 110, 13–32. 27475903 10.1016/j.plaphy.2016.07.018

[39] van der Ent A. , Przybylowicz W. J., de Jonge M. D., Harris H. H., Ryan C. G., Tylko G., Paterson D. J., Barnabas A. D., Kopittke P. M., Mesjasz-Przybylowicz J., X-ray elemental mapping techniques for elucidating the ecophysiology of hyperaccumulator plants, New Phytol., 2018, 218 (2), 432–452. 28994153 10.1111/nph.14810

[40] Matsuyama S. , Shimura M., Fujii M., Maeshima K., Yumoto H., Mimura H., Sano Y., Yabashi M., Nishino Y., Tamasaku K., Elemental mapping of frozen-hydrated cells with cryo-scanning X-ray fluorescence microscopy, X-Ray Spectrom., 2010, 39 (4), 260–266.

[41] Pushie M. J. , Pickering I. J., Korbas M., Hackett M. J., George G. N., Elemental and chemically specific X-ray fluorescence imaging of biological systems, Chem. Rev., 2014, 114 (17), 8499–8541. 25102317 10.1021/cr4007297 PMC4160287

[42] Srivastava M. , Ma L. Q., Rathinasabapathi B., Srivastava P., Effects of selenium on arsenic uptake in arsenic hyperaccumulator *Pteris vittata* L, Bioresour. Technol., 2009, 100 (3), 1115–1121. 18823776 10.1016/j.biortech.2008.08.026

[43] Kitajima N. , Kashiwabara T., Fukuda N., Endo S., Hokura A., Terada Y., Nakai I., Observation of arsenic transfer in leaf tissue of hyperaccumulator fern by utilizing synchrotron radiation micro-XRF imaging, Chem. Lett., 2008, 37 (1), 32–33.

[44] Fukuda N. , Kitajima N., Terada Y., Abe T., Nakai I., Hokura A., Visible cellular distribution of cadmium and zinc in the hyperaccumulator *Arabidopsis halleri* ssp. *gemmifera* determined by 2-D X-ray fluorescence imaging using high-energy synchrotron radiation, Metallomics, 2020, 12 (2), 193–203. 31845691 10.1039/c9mt00243j

[45] Taiz L. , Zeiger E., Plant Physiology, Sinaur Associates Inc, Sunderland, MA, 2002.

[46] H. , Marschner Mineral Nutrition of Higher Plants, 2nd edn, Academic Press Limited, London, UK, 1995.

[47] Tu C. , Ma L. Q., Effects of arsenic on concentration and distribution of nutrients in the fronds of the arsenic hyperaccumulator *Pteris vittata* L, Environ. Pollut., 2005, 135 (2), 333–340. 15734593 10.1016/j.envpol.2004.03.026

[48] Xu J.-Y. , Li H.-B., Liang S., Luo J., Ma L. Q., Arsenic enhanced plant growth and altered rhizosphere characteristics of hyperaccumulator *Pteris vittata*, Environ. Pollut., 2014, 194, 105–111. 25103044 10.1016/j.envpol.2014.07.017

[49] Schreiber L. , Hartmann K., Skrabs M., Zeier J., Apoplastic barriers in roots: chemical composition of endodermal and hypodermal cell walls, J. Exp. Bot., 1999, 50, 1267–1280.

[50] Assmann S. M. , Solute transport, in: Taiz L., Zeiger E., eds,. Plant Physiology, 4th ed. Sinauer, Sunderland, UK, 2006, pp. 95–129.

[51] Murphy A. S. , Bandyopadhyay A., Holstein S. E., Peer W. A., Endocytotic cycling of PM proteins, Annu. Rev. Plant Biol., 2005, 56 (1), 221–251. 15862095 10.1146/annurev.arplant.56.032604.144150

[52] Yang X. , Chen H., Dai X., Xu W., He Z., Ma M., Evidence of vacuolar compartmentalization of arsenic in the hyperaccumulator *Pteris vittata*, Chin. Sci. Bull., 2009, 54 (22), 4229–4233.

[53] Van Belleghem F. , Cuypers A., Semane B., Smeets K., Vangronsveld J., d'Haen J., Valcke R., Subcellular localization of cadmium in roots and leaves of *Arabidopsis thaliana*, New Phytol., 2007, 173 (3), 495–508. 17244044 10.1111/j.1469-8137.2006.01940.x

[54] Seregin I. , Kozhevnikova A., Roles of root and shoot tissues in transport and accumulation of cadmium, lead, nickel, and strontium, Russ. J. Plant Physiol., 2008, 55 (1), 1–22.

[55] Hokura A. , Omuma R., Terada Y., Kitajima N., Abe T., Saito H., Yoshida S., Nakai I., Arsenic distribution and speciation in an arsenic hyperaccumulator fern by X-ray spectrometry utilizing a synchrotron radiation source, J. Anal. Atom. Spectr., 2006, 21, 321–328.

[56] Danh L. T. , Truong P., Mammucari R., Foster N., A critical review of the arsenic uptake mechanisms and phytoremediation potential of *Pteris vittata*, Int. J. Phytoremediation, 2014, 16 (5), 429–453. 24912227 10.1080/15226514.2013.798613

[57] He Z. , Yan H., Chen Y., Shen H., Xu W., Zhang H., Shi L., Zhu Y. G., Ma M., An aquaporin PvTIP4;1 from *Pteris vittata* may mediate arsenite uptake, New Phytol., 2016, 209 (2), 746–761. 26372374 10.1111/nph.13637

[58] Wang X. , Ma L. Q., Rathinasabapathi B., Liu Y., Zeng G., Uptake and translocation of arsenite and arsenate by *Pteris vittata* L.: effects of silicon, boron and mercury, Environ. Exp. Bot., 2010, 68 (2), 222–229.

[59] Kashiwabara T. , Tanoi K., Kitajima N., Hokura A., Abe T., Nakanishi T., Nakai I., Comparative *in vivo* imaging of arsenic and phosphorus in *Pteris vittata* gametophyte by synchrotron μ-XRF and radioactive tracer techniques, Chem. Lett., 2019, 48 (4), 319–321.

